# Competitive fitness of asymptomatic bacteriuria *E. coli* strain 83972 against uropathogens in human urine

**DOI:** 10.1128/iai.00173-24

**Published:** 2024-05-23

**Authors:** Iris George, Manivannan Sivaperuman Kalairaj, Philippe E. Zimmern, Taylor H. Ware, Sargurunathan Subashchandrabose

**Affiliations:** 1 Department of Veterinary Pathobiology, College of Veterinary Medicine and Biomedical Sciences, Texas A&M University, College Station, Texas, USA; 2 Department of Biomedical Engineering, Texas A&M University, College Station, Texas, USA; 3 Department of Urology, University of Texas Southwestern Medical Center, Dallas, Texas, USA; 4 Department of Materials Science and Engineering, Texas A&M University, College Station, Texas, USA; Universite de Geneve, Geneva, Switzerland

**Keywords:** ABU 83972, UTI, UPEC, uropathogens, ABU, ELM

## Abstract

Urinary tract infection (UTI) is one of the most common bacterial infections worldwide. The main causative agent of UTI is uropathogenic *Escherichia coli* (UPEC). There is an immediate need for novel prophylactic and treatment strategies against UTI because of the increasing incidence of antimicrobial resistance among uropathogens. ABU 83972, an asymptomatic bacteriuria-causing *E. coli* strain*,* prevents UTI by suppressing the colonization of UPEC. However, the nature of competition and growth repression of UPEC by ABU 83972 is unclear and is the subject of our investigation. Here, we characterized the growth kinetics of ABU 83972 and uropathogens in human urine and laboratory media. Next, we performed a series of competitive co-culture experiments where ABU 83972 and uropathogens were inoculated at a 1:1 ratio in human urine and in various media, and their relative abundance was determined. In human urine, ABU 83972 outcompeted UPEC and additional uropathogens, reaching up to 90% of the total population after 24 hours of incubation. In contrast, UPEC outcompeted ABU 83972 in LB and M9 minimal media and exhibited superior colonization than ABU 83972 in the mouse urinary bladder. Since engineered living materials (ELMs) can be used to retain an organism of interest in a particular location, we developed ABU 83972-containing ELMs that effectively outcompeted UPEC in human urine. In summary, our work establishes that ABU 83972 outcompetes UPEC in a milieu- and cell-density-dependent manner, highlighting the importance of the metabolites and nutrients found in the human urine as determinants of the competitive fitness of ABU 83972.

## INTRODUCTION

Urinary tract infection (UTI) is a common bacterial infection, affecting approximately 150 million people each year globally (1). UTI begins upon the entry of bacteria in the urethra, from where they can ascend to the urinary bladder, causing bladder infection (cystitis). However, once in the bladder, these uropathogens can ascend to the kidneys triggering pyelonephritis, ultimately resulting in renal damage and/or dissemination to the blood (bacteremia/sepsis). Women are much more prone to UTIs than men, and 40% of women develop clinical UTI at least once in their lives (2). Clinically UTIs are classified as either uncomplicated or complicated UTI. Complicated UTIs occur in individuals with conditions like anatomical urinary tract abnormalities, compromised immune systems, renal transplant, pregnancy, or a history of recurrent UTI (2). Recurrent UTI is a major clinical problem that often leads to repeated and extended use of antibiotics that do not always prevent recurrence (3).

The main causative agent of UTI is uropathogenic *Escherichia coli* (UPEC), which is responsible for 65%–70% of all UTIs (4–6). Other clinically significant uropathogens include *Klebsiella pneumoniae*, *Staphylococcus saprophyticus*, *Enterococcus faecalis*, Group B *Streptococcus*, *Proteus mirabilis*, *Pseudomonas aeruginosa*, *Staphylococcus aureus, Acinetobacter baumannii,* and *Candida* spp. (5–11). Depending on the genetic composition of the *E. coli* strain and host factors, colonization of the urinary tract could result in UTI or asymptomatic bacteriuria (ABU). Genomes of UPEC strains encode major virulence factors such as adhesins (type 1 and P fimbriae) and toxins (hemolysin) (5, 12, 13). However, the presence of ABU with *E. coli* strains in the urinary tract does not cause any clinical symptoms or inflammatory response. Defective adherence of ABU *E. coli* strains to the urothelium due to mutations in the fimbrial genes has been reported as a critical factor in the attenuation of these strains (14–16).

Long-term colonization of ABU *E. coli,* specifically strain ABU 83972, can persist from months to years without evidence of symptoms and is reported to prevent colonization by uropathogens in the urinary tract (16–22). Deliberate colonization of this strain lowered the incidence of UTI in patients with neurogenic bladder related to spinal cord injury (16, 20). ABU 83972 has emerged as a safe and effective preventative approach against UTI. Previous studies have shown that this strain can compete against UPEC in pooled human urine (23, 24). However, the nature of that competition between ABU 83972 and uropathogens is not well understood and is the subject of this investigation.

Engineered living materials (ELMs) that integrate living microorganisms into biomaterial matrices could be used to precisely deliver microbes. The living components are typically bacteria or yeast, whereas the non-living components are made of natural or synthetic hydrogels (25, 26). The hydrogel supports the survival and growth of living cells by facilitating the diffusion of water, nutrients, gases, and biomolecules (27). The ELMs in these aspects resemble an engineered biofilm. We have observed that ELMs release living cells over sustained periods of time due to cell proliferation within the material (28). Here, we evaluated whether ABU 83972 embedded within an ELM could outcompete UPEC. In this study, we address critical questions on the competitive fitness of ABU 83972 against uropathogens by investigating the role of milieu, cell density, continuous replenishment of nutrients, colonization and relative fitness in the murine urinary tract, and release from ELMs.

## MATERIALS AND METHODS

### Bacterial strains and culture conditions

Bacterial strains used in this study are listed in Table 1. 10^10^ CFU/mL of ABU 83972 was cultured on LB plates containing rifampin 100 µg/mL and a spontaneous rifampin-resistant mutant was isolated. The isolate was verified as *E. coli* by MALDI-TOF, a mutation in the *rpoB* gene was confirmed by Sanger sequencing, and it exhibited identical growth patterns in LB and human urine to the wild-type ABU 83972. The rifampin-resistant ABU 83972 was used in the coculture assays. Filter-sterilized human urine (Cone Bioproducts, Seguin, TX, USA) that was pooled from 10 adult female donors who had no history of UTI or diabetes was purchased. Two different lots of filter-sterilized human urine purchased 2 years apart were used in our studies. Artificial urine media (AUM) (29) and M9 minimal media as described in reference (30) but containing glycerol instead of glucose were also used in competition assays. Strains were grown in LB broth with or without 1.5% agar (Fisher bioreagent, Pittsburgh, PA, USA) and tryptic soy broth (Beckton Dickinson, Franklin lakes, NJ, USA) with or without 1.5% agar (for *S. aureus*). All strains were grown in LB or TSB without antibiotics at 37°C for 24 hours aerobically at 200 RPM unless otherwise noted. For determining the growth characteristics, overnight cultures were diluted 100-fold and 1,000-fold in LB and transferred to 96-well plates. Overnight cultures were resuspended in PBS before diluting 100-fold and 1,000-fold in pooled human urine for growth assays. The OD_600_ was measured every hour until 6 hours (Cytation 5 plate reader, Agilent Biotek). Generation time was calculated using R studio (31).

**TABLE 1:** Bacterial strains used in this study

Strain	Description^*a*^	Source
ABU 83972	ABU *E. coli*	(22)
ABU 83972 Rif^R^	Rifampin-resistant *E. coli*	Subash lab
CFT073	Wild-type UPEC	(32)
CFT073 Rif^R^	Rifampin-resistant *E. coli*	Mobley lab
CFT073 Kan^R^	Kanamycin-resistant *E. coli*	(33)
UTI89	Wild-type UPEC	(34)
KP4 Rif^R^	Rifampin-resistant *K. pneumoniae*	(35)
ATCC 17978	Wild-type *A. baumannii*	ATCC
ATCC 27853	Wild-type *P. aeruginosa*	ATCC
SF 8300	Methicillin-resistant *S. aureus*	(36)

^*a*^ Rif^R^, rifampin resistant; Kan^R^, kanamycin resistant.

### PCR verification of ABU 83972

PCR was carried out using the primers specific to the plasmid pABU found in ABU 83972 with the primers 5′CGAAGGACAAGCAGGGAGTT3′ and 5′AAGCTCTCAGACGCACGAAA3′. PCR products were separated in a 1% agarose gel, stained with ethidium bromide, and visualized using a BioRad Chemidoc MP system.

### Competition of ABU 83972 against uropathogens

For competition experiments, overnight cultures of ABU 83972 Rif^R^ and UPEC CFT073 were resuspended in PBS before adjusting OD_600_ = 1, mixed in a 1:1 ratio, and diluted to various concentrations indicated in the figure legends in pooled human urine or LB. Antibiotics were not added to the coculture assays throughout the study. At the beginning and after 24 hours of incubation, samples were diluted and plated on LB agar with and without rifampin and the colony-forming unit (CFU/mL) was determined. To ensure that antibiotic resistance markers did not affect competition results, strains with other antibiotic markers, such as rifampin or kanamycin resistance, or without any markers (Fig. 2) were used in competition assays. We also tested the growth of all strains on selective plates to ensure that only resistant strains grew in the presence of appropriate antimicrobial agents. Competition of ABU 83972 with multiple uropathogens (Table 1) was determined in both pooled human urine and LB (TSB for *S. aureus*). We also conducted a competition experiment between ABU 83972 Rif^R^ and CFT073, varying their beginning ratios from 1:1 and the number of cells in the combination inoculum. In addition, the competition pattern of the co-culture was examined in M9 minimal medium and artificial urine medium. We also performed a competition experiment in pooled human urine containing ABU 83972 Rif^R^ and CFT073 in a 1:1 ratio that was incubated under anaerobiosis (Bactron 300, Sheldon Manufacturing). Additionally, a competition experiment between ABU 83972 Rif^R^ and CFT073 in a 1:1 ratio was conducted in the filter-sterilized supernatant of an overnight culture of ABU 83972 Rif^R^, CFT073, and coculture of ABU 83972 Rif^R^ and CFT073 in pooled human urine.

### Competition under a continuous flow of pooled human urine

Pooled human urine with a mixed inoculum of ABU 83972 Rif^R^ and UPEC CFT073, prepared as described above, was added with pooled human urine using a syringe pump (Braintree Scientific, USA) at a flow rate of 50 µL/minute for 10 hours. Every 2 hours, 6 mL of culture was removed and maintained at the initial volume of the co-culture inoculum constant, and CFU/mL was calculated on LB with and without rifampin. Competition experiments were conducted simultaneously without the addition of pooled human urine as controls.

### Mouse colonization studies

The mouse model (CBA/J) of urinary tract colonization was used with individual or mixed inocula as previously described (37, 38). Bacterial strains were cultured overnight in LB with shaking, adjusted to an OD_600_ of 3, and used as individual or mixed (1:1) inocula. Female CBA/J mice that were ~6 weeks old were used in our study. Briefly, 10^8^ CFUs of ABU 83972 Rif^R^ + UPEC CFT073 (1:1), ABU 83972 + UPEC CFT073 Rif^R^ (1:1), ABU 83972 Rif^R^ (alone), and UPEC CFT073 (alone) were inoculated in the urinary bladder (*N* = 5 mice/group). Mice were randomly assigned to groups normalized for body weight. The sample size was determined based on our prior report on the murine model of colonization with ABU 83972 and UPEC CFT073 (37). At 24 hours after inoculation, urine samples were collected, and mice were euthanized. Urine collection was performed by scruffing and briefly holding mice over sterile surfaces. Vaginal lavage was collected by infusing 50 µL PBS in the vagina after euthanasia (39). Organs were aseptically removed, homogenized, and plated on LB agar with or without rifampin to determine viable count. A separate mouse experiment was also performed with an individual inoculum of ABU 83972 Rif^R^ and UPEC CFT073 (*N* = 5 mice/group). Mice were transurethrally infected with individual inoculum, and urine was collected for 5 days. On the fifth day after inoculation, mice were euthanized, organs were removed, homogenized, and plated on LB agar with and without antibiotics for quantitative colony counts. Cages of mice inoculated with different strains were handled separately to prevent cross-contamination. Bacterial load was the primary outcome measure in our experiments. Inclusion and exclusion criteria were not set *a priori,* and data from all 25 mice are presented in Fig. 7 and Fig. S7. Operators were aware of experimental groups during the study to ensure no cross-contamination between cages/groups. Data were analyzed by the Mann-Whitney test in GraphPad Prism. Mouse urine from mice aged 4–8 weeks was collected, pooled, and filter sterilized for use in the *in vitro* competition assay between ABU 83972 Rif^R^ and UPEC CFT073.

### Fabrication of engineered living material

The engineered living materials were made by free radical polymerization of 2-hydroxyethyl acrylate (HEA) monomer and *N,N′*-methylenebisacrylamide (BIS) crosslinker with 1 × 10^11^ CFU/mL of ABU 83972 Rif^R^. The pre-gel solution was prepared by mixing 10 wt% of HEA, 0.1 wt% of BIS, 0.04 wt% of lithium phenyl-2,4,6-trimethylbenzoylphosphinate, 6.86 wt% of distilled water, 23 wt% of LB, and 1 × 10^11^ CFU/mL of ABU 83972 Rif^R^ (60 wt%). This pre-gel solution was subsequently placed in a PE50 catheter (Braintree Scientific) and exposed to 365 nm UV irradiation (UVP Crosslinker CL-3000, Analytik Jena) at 1.2 mW/cm^2^ intensity for 2 minutes to trigger polymerization. The polymerized ELMs were dispensed from the catheter with a sterile 27G needle, trimmed to 4 mm length with a razor blade, and washed three times in PBS. The resulting ELMs had a volume of ~1 µL.

### ABU 83972 release from ELM and competition with UPEC

The fabricated ELMs with ABU 83972 Rif^R^ were incubated in 2 mL of pooled human urine at 37°C and 200 RPM. The ELMs were removed from the pooled human urine every 24 hours, washed three times in PBS by vortexing to remove bacteria on the surface of ELMs, transferred to 2 mL of pooled human urine, and incubated under the same conditions. This procedure was continued for 3 days. ABU 83972 Rif^R^ released from the ELMs was quantified after 2 hours of incubation in pooled human urine by viable counts, as described (28). These 3-day-grown ELMs were used for competition experiments. Before adding UPEC CFT073 for the competition, 3-day-old ELMs were incubated in fresh pooled human urine for 2 hours to mimic a prophylactic setting where ABU 83972 is present prior to pathogen invasion. Every 24 hours, the CFU/mL of pooled human urine containing ABU-releasing ELMs and UPEC CFT073 was determined on LB agar, with and without antibiotics. Additionally, mock ELMs without ABU 83972 Rif^R^ were incubated with UPEC CFT073 in pooled human urine and CFU/ml of UPEC CFT073 was determined.

## RESULTS

### ABU 83972 and UPEC exhibit comparable growth in pooled human urine

**Fig 1:**
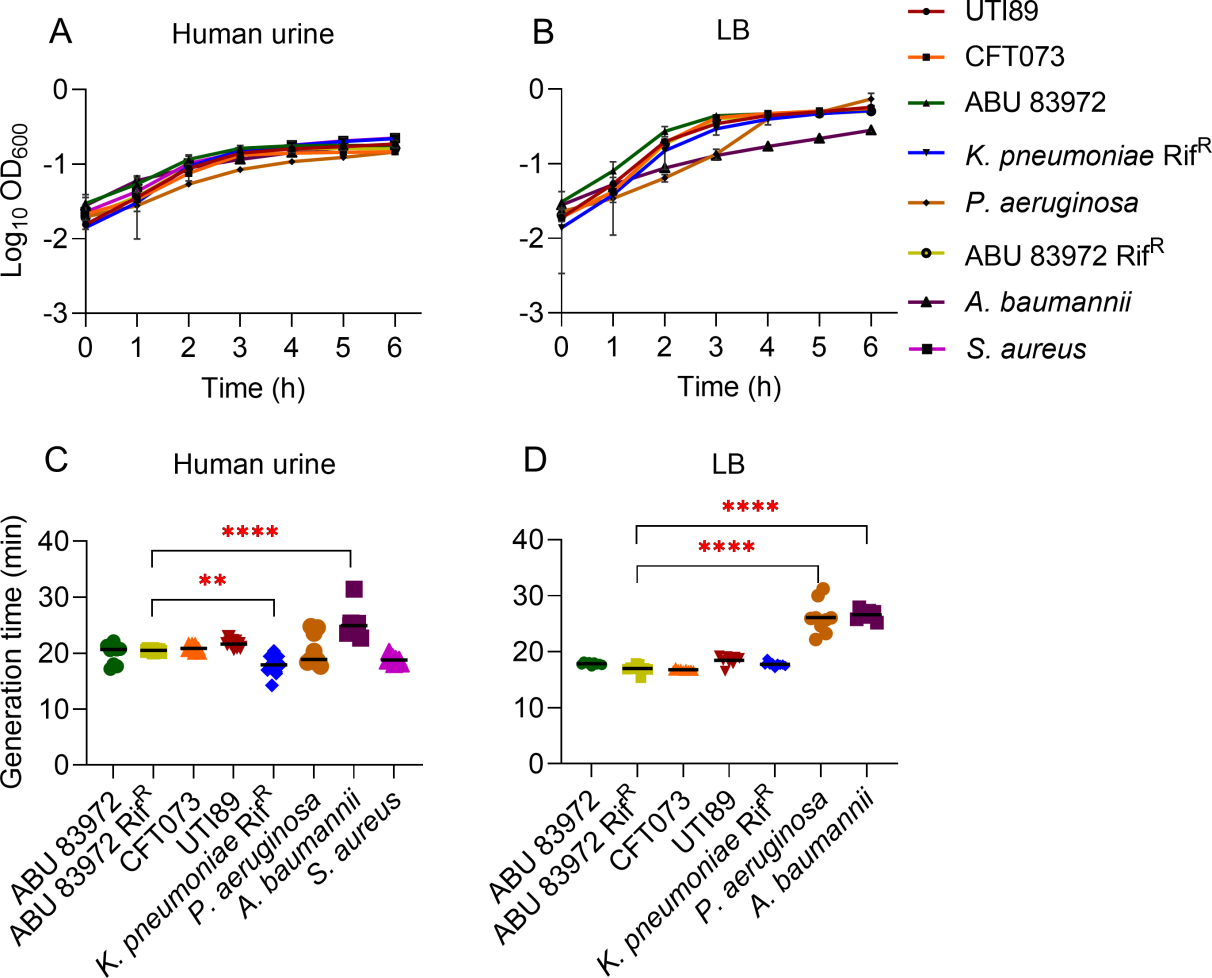
Growth of ABU 83972, UPEC, and other uropathogens in human urine. Growth of ABU 83972, UPEC strains CFT073 and UTI89, and other uropathogens was determined in human urine (**A**) and in LB medium (**B**). Mean ± standard deviation is depicted here. The doubling time of each strain in human urine (**C**) and in LB (**D**) during the exponential phase was determined using R Studio. Bars indicate the median. **P < 0.01 and ****P < 0.0001, ANOVA with Dunnett’s test.

ABU 83972 was verified by PCR using primers specific for the plasmid that is only present in ABU 83972 not in UPEC (Fig. S1). To understand the growth characteristics of each bacterial strain (Table 1), we inoculated them in pooled human urine and LB medium at 100-fold dilution and assessed their growth (OD_600_) over time. The growth patterns of *E. coli* strains and *K. pneumoniae* Rif^R^ were comparable in the exponential phase and transition to the stationary phase in both pooled human urine and LB after ~3 hours of incubation (Fig. 1A and B). Likewise, a 1:1,000 dilution of ABU 83972 and UPEC CFT073 exhibited comparable growth pattern in both pooled human urine and LB media (Fig. S2). *P. aeruginosa, S. aureus,* and *A. baumannii* also grew in pooled human urine in a pattern comparable with that of *E. coli* (Fig. 1A; Fig. S3). The generation times, calculated using R studio (31), revealed that the generation times of ABU 83972 and ABU 83972 Rif^R^ were identical in pooled human urine and comparable findings were observed in LB (Fig. 1C and D). Since they exhibited identical generation times, we used ABU 83972 Rif^R^ in further experiments to utilize the selective marker to obtain differential counts from co-cultures with uropathogens. Moreover, there was no significant difference in the generation time of ABU 83972 Rif^R^ and UPEC in both pooled human urine and LB (Fig. 1C and D). Among the uropathogens tested here, *K. pneumoniae* Rif^R^ had the shortest generation time in pooled human urine and was significantly different from ABU 83972 Rif^R^ (Fig. 1C). However, there was no significant difference between the doubling time of ABU 83972 Rif^R^ and *K. pneumoniae* Rif^R^ in LB (Fig. 1D). In addition, there was a notable increase in the generation time of *A. baumannii* compared to ABU 83972 Rif^R^ in both pooled human urine and LB (Fig. 1C and D). *P. aeruginosa* had significantly longer generation times in LB (Fig. 1D) but not in pooled human urine (Fig. 1C) when compared to ABU 83972 Rif^R^. These findings demonstrate that ABU 83972 and UPEC exhibit comparable growth in both pooled human urine and LB.

### ABU 83972 outcompetes UPEC during growth in pooled human urine

**Fig 2:**
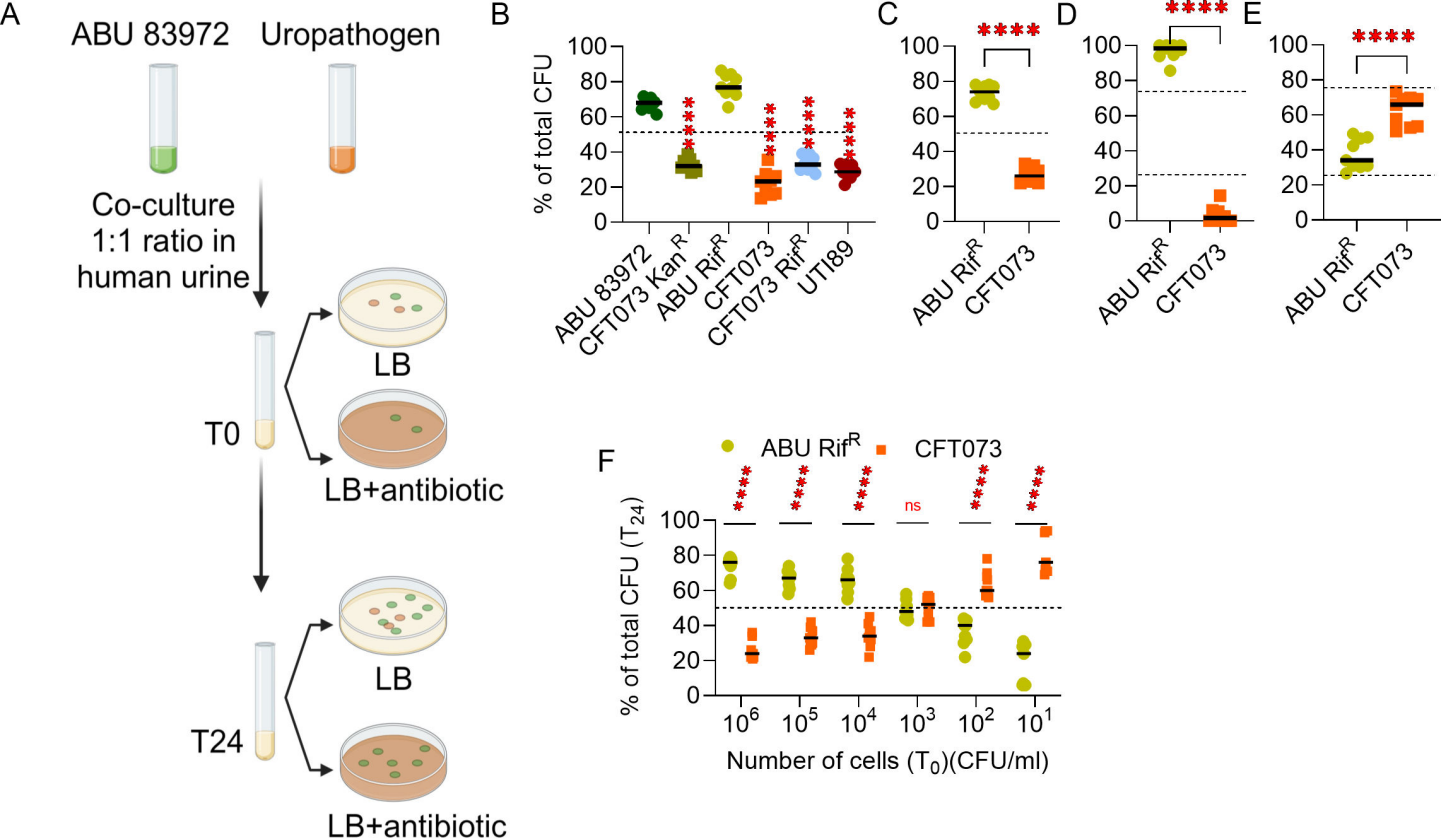
ABU 83972 outcompetes UPEC in pooled human urine. (**A**) Workflow of competitive co-culture experiments and bacterial enumeration. (**B**) Relative abundance of ABU 83972 and UPEC (CFT073 or UTI89) at 24 hours in pooled human urine with co-culture inocula in a 1:1 ratio (10^6^ CFU/mL of each strain). (**C**) Relative abundance of ABU 83972 Rif^R^ (ABU Rif^R^) and UPEC CFT073 under anaerobic conditions. The level of competition of ABU 83972 was also determined by altering the initial ratio of ABU 83972 Rif^R^ to CFT073 in human urine to 3:1 (**D**) and 1:3 (**E**). (**F**) Relative abundance of ABU 83972 Rif^R^ and CFT073 mixed in a 1:1 ratio in the inocula at various cell densities (10^6^ to 10^1^ CFU/mL) and cultured in human urine for 24 hours. The dashed line represents the initial population of the strains (50% B, C, and F and 25%/75% D and E). Bars indicate the median. ****P < 0.0001, *t*-test. Rif^R^, rifampin resistant; and Kan^R^, kanamycin resistant.

ABU 83972 and other uropathogens grew in pooled human urine when inoculated individually (Fig. 1A and C). We assessed the competitive fitness of ABU 83972 by co-culturing this strain with uropathogens in pooled human urine (Fig. 2A). Differential plate counts with antibiotic-containing and -free media were used to enumerate bacterial counts at T0 and T24. Co-cultures were adjusted to contain competing strains at a 1:1 ratio (50% of the population each) at the beginning of incubation at 10^6^ CFU/mL of each strain. To ensure that the competition is not affected because of antibiotic selection, we tested the following combinations: ABU 83972 vs CFT073 Kan^R^; ABU 83972 Rif^R^ vs CFT073; and CFT073 Rif^R^ vs ABU 83972. In all these combinations, ABU 83972 significantly outcompeted UPEC CFT073, indicating that the competitive nature of ABU 83972 is not dependent on the presence of selection markers (Fig. 2B). Additionally, ABU 83972 Rif^R^ successfully competed against UTI89, another prototypical UPEC strain (Fig. 2B). In addition, the competition experiment was performed for up to 96 hours to determine the temporal changes in the relative abundance of ABU 83972 during co-culture with UPEC. ABU 83972 significantly outcompeted UPEC in pooled human urine at later time points up to 96 hours (Fig. S4). Furthermore, we investigated the potential role of oxygen in the competitive nature of ABU 83972 by incubating the cocultures under anaerobic conditions. Similar to aerobic conditions, ABU 83972 significantly outcompeted against UPEC CFT073 under anaerobiosis (Fig. 2C). Collectively, ABU 83972 has a significant competitive advantage over UPEC in pooled human urine regardless of oxygen availability.

### ABU 83972 competition with UPEC is affected by inoculum ratio and cell density

**Fig 3:**
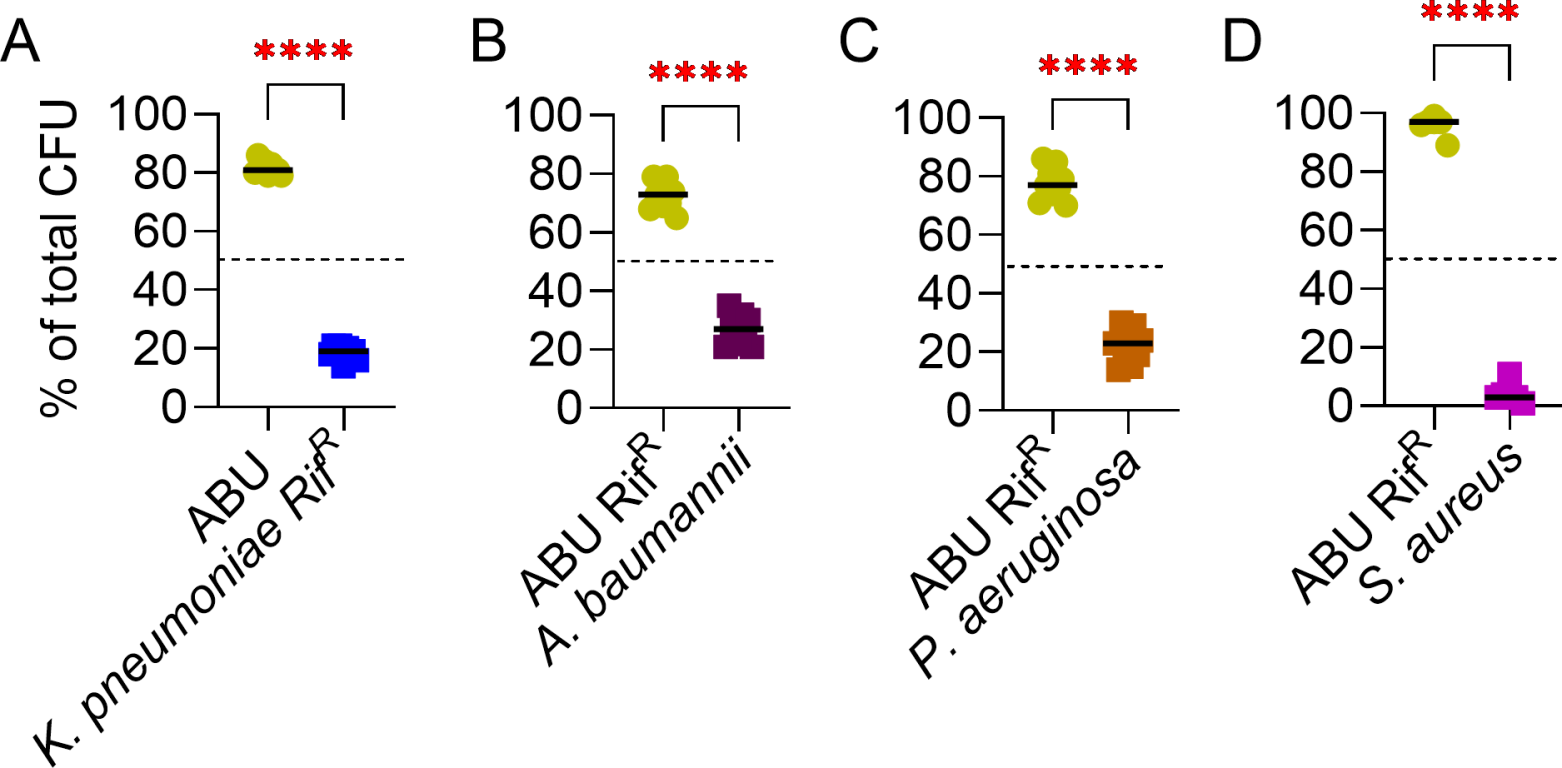
ABU 83972 outcompetes clinically important non-UPEC uropathogens in pooled human urine. Relative abundance of ABU 83972 (ABU) or ABU 83972 Rif^R^ (ABU Rif^R^) and *K. pneumoniae* Rif^R^ (**A**), *A. baumannii* (**B**), *P. aeruginosa* (**C**), and *S. aureus* (**D**) at 24 hours in pooled human urine with co-cultures inoculated in a 1:1 ratio. The dashed line represents the initial population of the strains (50%). Bars indicate the median. ****P < 0.0001, *t*-test.

Next, we asked how the initial abundance of ABU 83972 impacts the competition against UPEC in pooled human urine. This was determined by altering ABU 83972 and UPEC ratios at T0 in co-cultures from 1:1 (Fig. 2B) to 3:1 and 1:3, respectively (Fig. 2D and E). When ABU 83972 was present at a higher concentration at T0 (75%), it eventually reached ~100% by 24 hours, whereas UPEC population declined from 25% to less than 10% (Fig. 2D). When ABU 83972 and UPEC were mixed in a 1:3 ratio, the ABU 83972 load increased significantly from 25% at T0 to 35% at T24, with a concurrent decrease in the UPEC abundance from 75% to 65% (Fig. 2E). Furthermore, we tested the role of cell density in ABU 83972 competition with UPEC by starting co-culture experiments at a 1:1 ratio but with total bacterial cells ranging from 10^6^ through 10^1^ CFU/mL in pooled human urine. ABU 83972 successfully outcompeted UPEC when the bacterial load at T0 was 10^6^ through 10^4^ CFU/mL (Fig. 2F). Notably, ABU 83972 and UPEC exhibited identical fitness when inoculated at 10^3^ CFU/mL. However, UPEC significantly outcompeted ABU 83972 when the initial bacterial load was 100 CFU/mL or lower (Fig. 2F). These results reveal that ABU 83972 can prevent UPEC growth even when they are found at a lower concentration than UPEC in pooled human urine in a cell density-dependent manner.

### ABU 83972 outcompetes a diverse range of uropathogens

To explore the competitive interactions of ABU 83972 with clinically important non-UPEC uropathogens, we performed competition assays between ABU 83972 and uropathogens such as *K. pneumoniae* Rif^R^*, A. baumannii, P. aeruginosa,* and *S. aureus* in pooled human urine. There was a significant decrease in *K. pneumoniae* Rif^R^*, A. baumannii, P. aeruginosa,* and *S. aureus* as a percentage of total population compared to ABU 83972 at T24 (Fig. 3A through D) Specifically, ABU 83972 predominated during co-cultures with *K. pneumoniae* Rif^R^ and *S. aureus* by expanding to 81% and 97% of the total population respectively (Fig. 3A and D). Collectively, our results indicate that ABU 83972 has a significant competitive advantage against multiple clinically important uropathogens.

### UPEC outcompetes ABU 83972 in laboratory media but not in artificial urine medium

**Fig 4:**
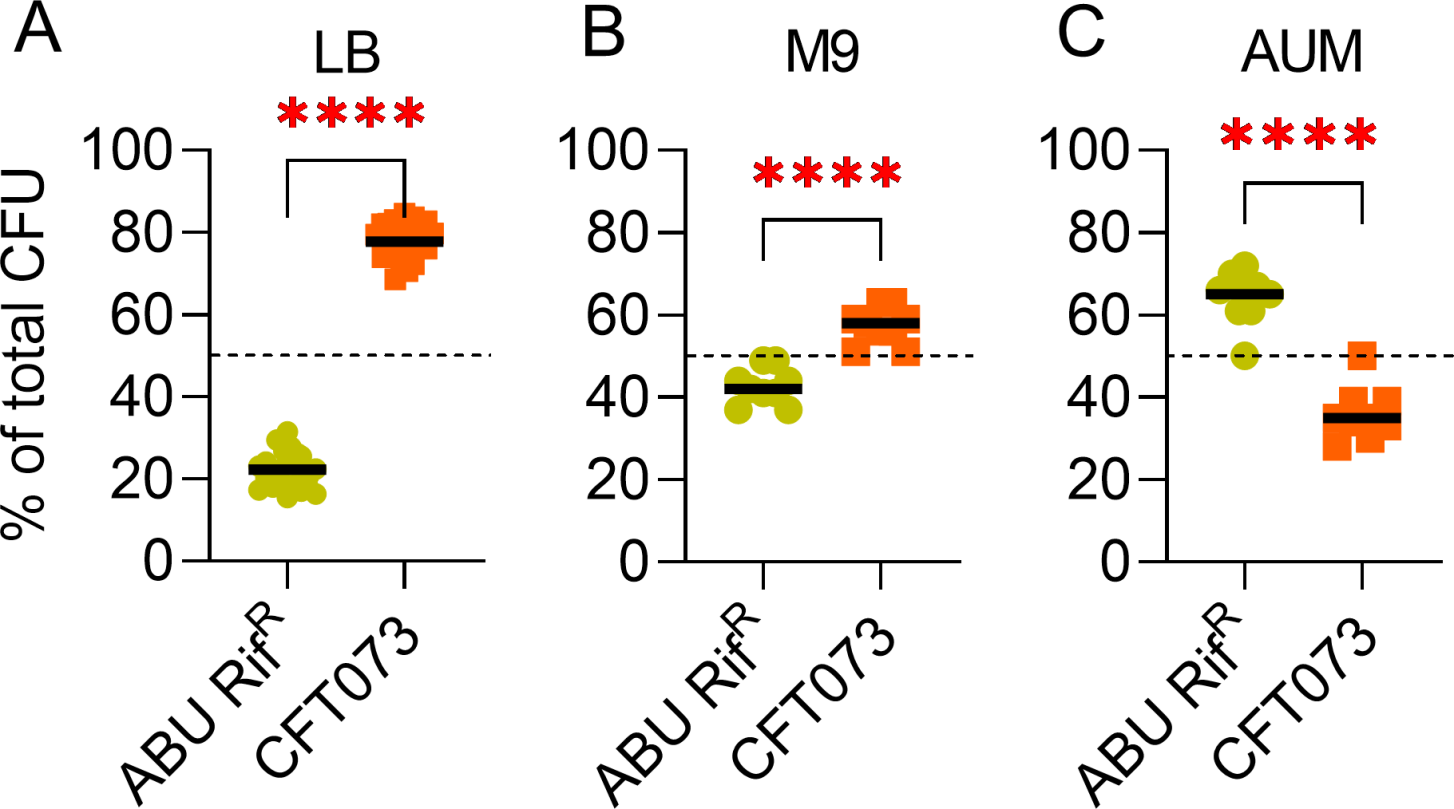
ABU 83972 retains competitive advantage against UPEC in artificial urine medium. Relative abundance of bacteria after 24 hours of incubation in co-cultures of ABU 83972 Rif^R^ with CFT073 in LB (**A**), M9 minimal medium (**B**), and AUM-artificial urine medium (**C**) started at a 1:1 ratio in the inocula (10^6^ CFU/mL of each strain). The dashed line represents the initial population of the strains. Bars indicate the median. ****P < 0.0001, *t*-test.

Since ABU 83972 outcompetes uropathogens in pooled human urine, we investigated the role of nutrient composition in driving this competition. We performed competitive growth assays in a nutrient-rich medium (LB), a minimal medium (M9), and an AUM. ABU 83972 notably failed to compete with UPEC, and UPEC expanded to ~75% of the entire population compared to 50% at T0 in LB (Fig. 4A). UPEC strain UTI89 and *K. pneumoniae* Rif^R^ also significantly outcompeted ABU 83972 during co-culture in LB (Fig. S5A and B). Nevertheless, ABU 83972 outcompeted effectively against *A. baumannii* and *P. aeruginosa* to varying degrees in LB (Fig. S5C and D). Because TSB is more suitable for *S. aureus*, we used TSB instead of LB as a rich medium for competition assays. ABU 83972 outcompeted *S. aureus* by expanding to more than 95% in TSB (Fig. S5E). Furthermore, we assessed the antagonism between ABU 83972 and UPEC in the M9 minimal medium. UPEC outcompeted ABU 83972 in M9 minimal medium but to a lesser extent than in LB (Fig. 4B). We also tested the competition in artificial urine medium since variability, both interpersonal and in the same individual over time, is a known limitation of using human urine for bacterial culture (40). ABU 83972 successfully competed against UPEC in AUM and accounted for 65% of the overall population after 24 hours of incubation (Fig. 4C). Although ABU 83972 antagonized UPEC in AUM, the magnitude was lower compared to pooled human urine (Fig. 2B and 4C). These results, taken in light of the competition assays in human urine, highlight the potential role of nutrients in human urine in determining the outcome of competitive growth between ABU 83972 and UPEC.

### Addition of nutrients has minimal impact on the extent of ABU 83972 competition against UPEC in pooled human urine

**Fig 5:**
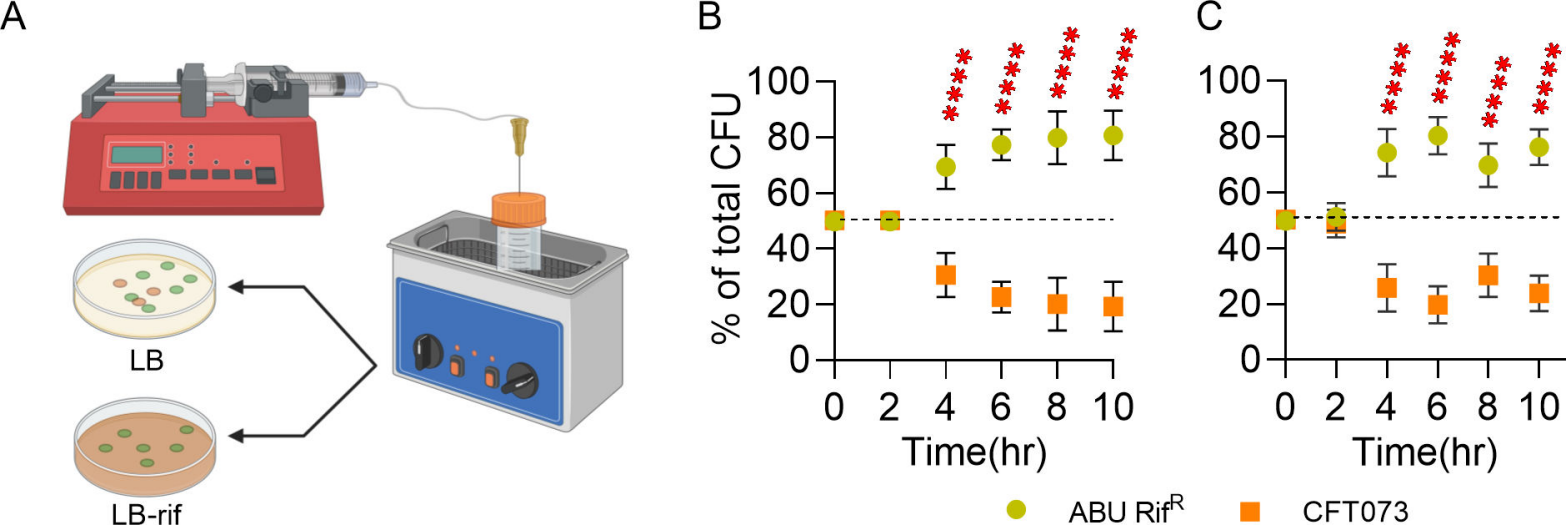
Nutrient availability does not affect the competitive fitness of ABU 83972 in human urine. Schematic representation of competition assay under the continuous flow of pooled human urine (**A**). Relative abundance of ABU 83972 Rif^R^ and CFT073 inoculated at a 1:1 ratio (10^6^ CFU/mL of each strain) in human urine was determined in pooled human urine (50 µL/min) added cultures (**B**) and batch cultures (**C**). Mean and standard deviation are depicted. The dashed line represents the initial population of the strains. ****P < 0.0001, *t*-test.

**Fig 6:**
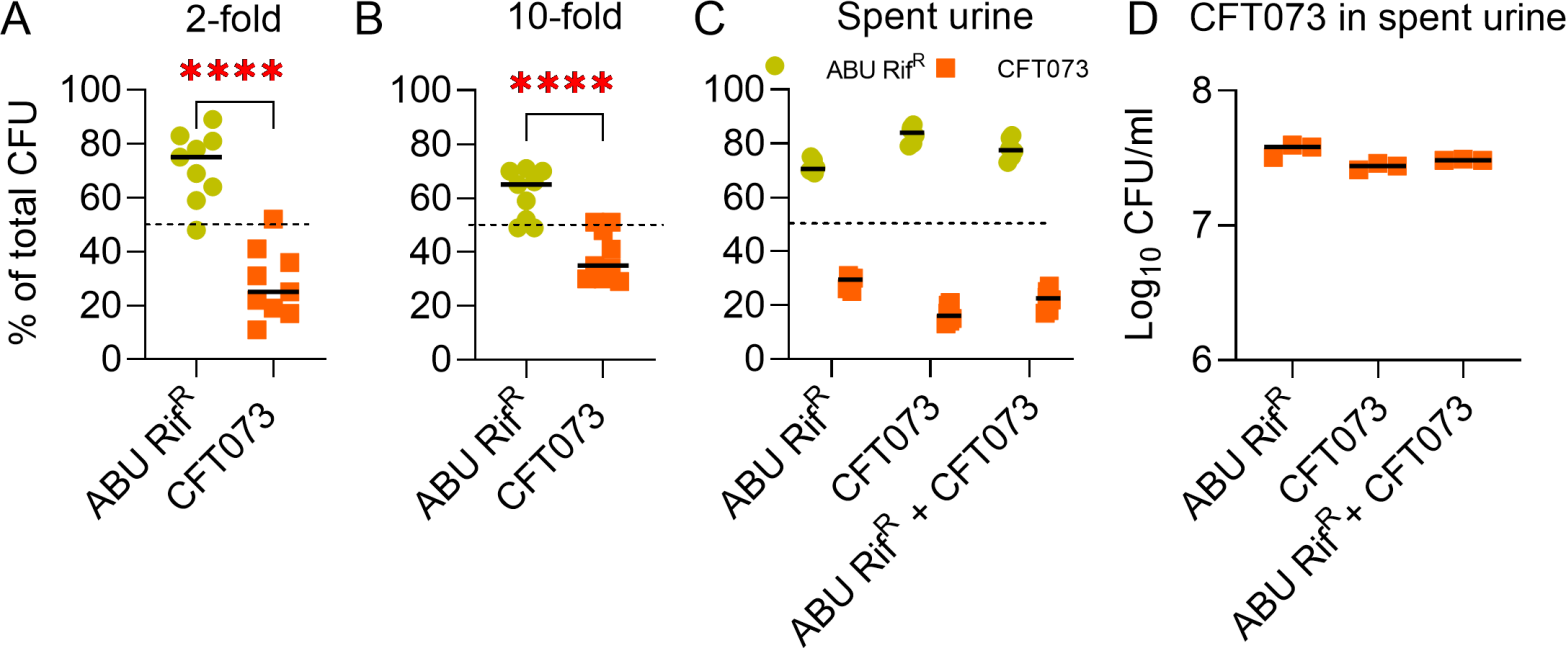
ABU 83972 outcompetes against UPEC during nutrient starvation. Relative abundance of ABU 83972 Rif^R^ and UPEC CFT073 was determined in human urine diluted 2-fold (**A**) or 10-fold (**B**) with sterile water and in spent pooled human urine from cultures of ABU 83972 or CFT073 or ABU 83972 and CFT073 (**C**). Growth of CFT073 in spent pooled human urine from cultures of ABU 83972 or CFT073 or ABU 83972 and CFT073 (**D**) after 24 hours. The dashed line represents the initial population of the strains (**A–C**). Bars indicate the median. ****P < 0.0001, *t*-test.

Competition assays were performed to mimic, at least in part, the continuous flow of urine from the kidneys to the urinary bladder by adding co-cultures of ABU 83972 and UPEC in human urine with pooled human urine (50 µL/minute) (Fig. 5A). ABU 83972 emerged as the dominant strain against UPEC by 4 hours and peaked at 10 hours by expanding to 80% of the total population (Fig. 5B), which was comparable to our results at 24 hours (Fig. 2B). A control co-culture without the addition of pooled human urine also showed an identical competition pattern between ABU 83972 and UPEC (Fig. 5C). Next, we tested whether nutrient depletion influences the competitive fitness of ABU 83972 in human urine. This was determined by repeating the competition experiments in 2-, and 10-fold-diluted human urine. There was a significant increase in the relative abundance of ABU 83972 in 2- and 10-fold-diluted human urine (Fig. 6A and B). However, the magnitude of change in relative abundance is lower between ABU 83972 and UPEC CFT073 in 10-fold-diluted human urine compared to undiluted urine (Fig. 2B and 6B). We have also assessed the growth rate of ABU 83972 and CFT073 in diluted pooled human urine and found that both cultures exhibited similar growth patterns in 2-fold- and 10-fold-diluted human urine (Fig. S6). These findings reveal that the concentration of nutrients in the human urine does not affect the competitive fitness of ABU 83972 relative to UPEC and that ABU 83972 continues to compete with UPEC during nutrient starvation in urine.

### ABU 83972 outcompetes UPEC in spent pooled human urine

Next, we investigated whether the competitive advantage of ABU 83972 is dependent on the selective depletion of nutrients in the urine that are required for the optimal growth of UPEC and/or secretion of inhibitory metabolites by ABU 83972 against UPEC. This was determined by conducting a competition experiment between ABU 83972 and UPEC CFT073 (1:1 ratio) in spent supernatants from the cultures of ABU 83972 or UPEC or ABU 83972 and UPEC in pooled human urine. Remarkably, ABU 83972 significantly outcompeted UPEC in all the scenarios, dominating by 71%, 84%, and 78% in the spent supernatant from the cultures of ABU 83972, UPEC, and ABU 83972 and UPEC, respectively (Fig. 6C). The remaining population comprised UPEC, and UPEC exhibits comparable growth in its own spent supernatant and that from ABU 83972 and ABU 83972 and UPEC co-cultures (Fig. 6C and D). Our results indicate that ABU 83972 did not appear to release any inhibitory substances that could hinder the growth of UPEC (Fig. 6D). Collectively, these findings reveal that ABU 83972 maintains its competitive advantage over UPEC in spent pooled human urine.

### ABU 83972 does not compete with UPEC in the murine urinary tract

**Fig 7:**
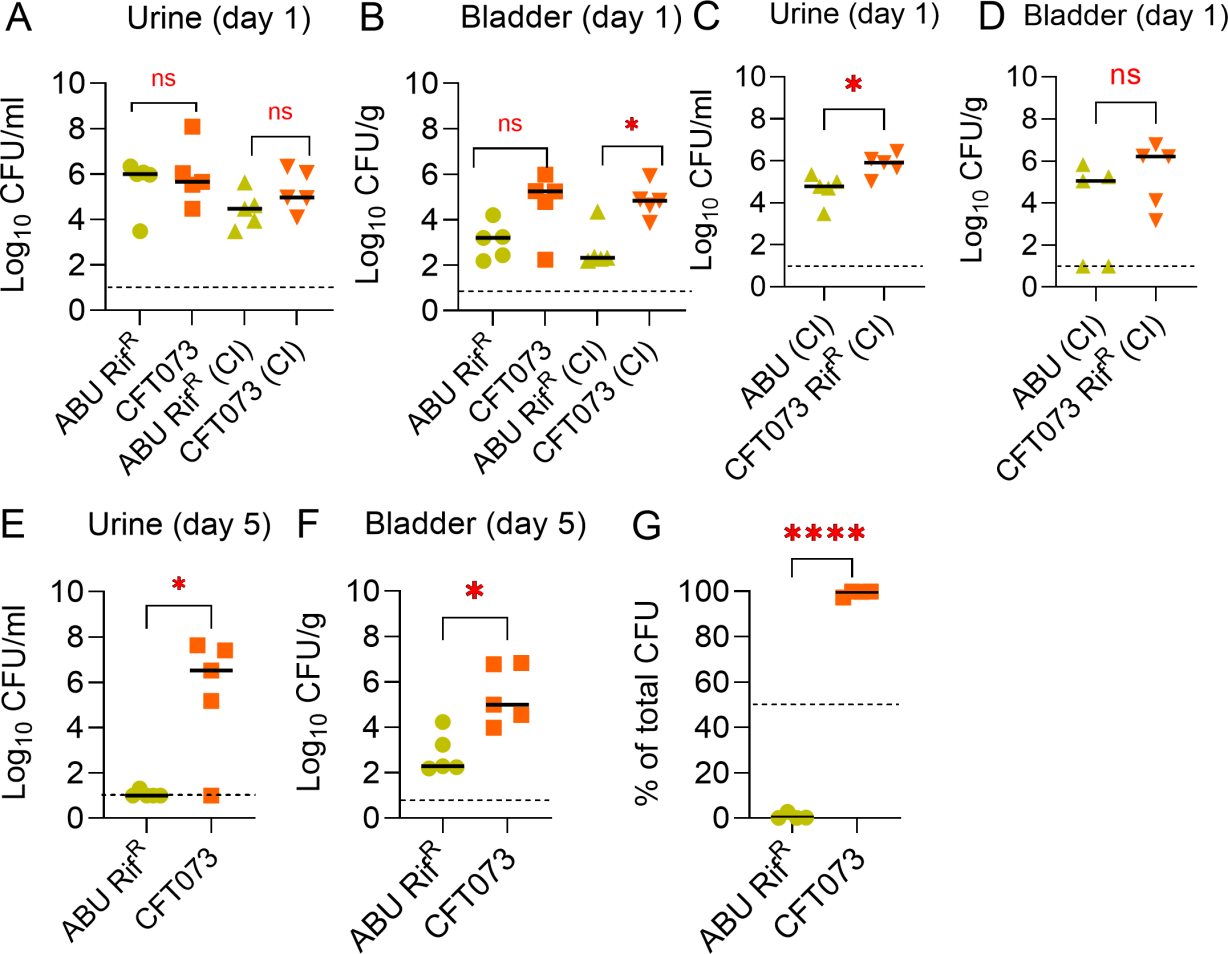
UPEC colonizes better than ABU 83972 in the murine urinary tract. The bacterial load in urine (**A**) and bladder (**B**) of mice transurethrally inoculated with a 1:1 mixture of ABU 83972 Rif^R^ and CFT073 (co-inoculation, CI), ABU 83972 Rif^R^ alone, or CFT073 alone at 1-day post-inoculation. The number of bacteria present in the urine (**C**) and bladder (**D**) of mice inoculated transurethrally with a 1:1 mixture of ABU 83972 and CFT073 Rif^R^. Bacterial burden in urine (**E**) and bladder (**F**) of mice inoculated with either ABU 83972 Rif^R^ or CFT073 at 5 days post-inoculation. Each symbol represents a mouse. Dotted line indicates the limit of detection (10 CFU/mL or g). *P < 0.05, Mann-Whitney test. (**G**) Relative abundance of ABU 83972 Rif^R^ and CFT073 inoculated at a 1:1 ratio in mouse urine *in vitro*. Bars indicate the median. The dashed line represents the initial population of the strains. ****P < 0.0001, *t*-test.

Our *in vitro* results clearly indicate the antagonism of ABU 83972 toward UPEC in human urine. To investigate the competitive fitness of ABU 83972 *in vivo*, female CBA/J mice were inoculated with either ABU 83972, UPEC CFT073, or a mixed inoculum containing equal numbers of both strains. Bacterial loads in urine and tissues were determined after 24 hours. ABU 83972 exhibited a lower fitness relative to UPEC in the urinary bladder and vaginal lavage (Fig. 7B; Fig. S7B) during co-inoculation with UPEC. There was no significant difference between ABU 83972 and UPEC loads in urine, other urogenital organs, and systemic sites (spleen and liver) (Fig. 7A; Fig. S7). To further validate our results from mouse colonization experiments, we repeated the *in vivo* competition by using the parental strain of ABU 83972 and a rifampin-resistant UPEC CFT073. Regardless of the presence or absence of rifampin resistance, ABU 83972 failed to compete against UPEC in the murine urinary tract (Fig. 7A through D). UPEC CFT073 Rif^R^ significantly outcompeted ABU 83972 in mouse urine at 24 hours postinfection (Fig. 7C). UPEC exhibited higher relative fitness during coinfection with ABU 83972 in the bladder, other urogenital organs, and systemic sites (spleen and liver), but this difference was not statistically significant (Fig. 7D; Fig. S7G through L). To further investigate the colonization of the murine urinary tract by ABU 83972, we administered these strains individually into the bladder of mice, and colonization was allowed to progress for 5 days. The load of ABU 83972 in mouse urine and bladders decreased dramatically as compared to UPEC (Fig. 7E and F). Similar trends of decreased colonization that were not statistically significant were also observed in other organs on the fifth day of infection (Fig. S7M through R). To probe the discrepancy between our findings in human urine and mouse colonization, we performed an *in vitro* competition experiment between ABU 83972 and UPEC CFT073 in mouse urine. Consistent with our *in vivo* findings, UPEC successfully outcompeted against ABU 83972 accounting for >97% of the total population in mouse urine at T24 (Fig. 7G). In summary, UPEC exhibits superior colonization of the murine urinary tract and mouse urine *in vitro* compared to ABU 83972.

### ABU 83972 released from engineered living materials prevents UPEC expansion

**Fig 8:**
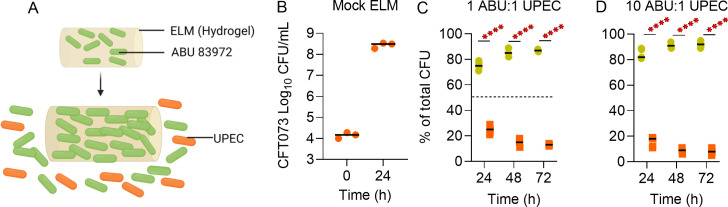
ABU 83972 released from ELM significantly decreases the UPEC load in human urine. (**A**) ABU 83972 Rif^R^ proliferation within ELM increases its volume and releases ABU 83972 Rif^R^ which then competes against UPEC. (**B**) UPEC CFT073 load in the presence of a mock ELM (not encapsulated with ABU 83972) in pooled human urine. Relative abundance of ABU 83972 Rif^R^ and CFT073 in human urine with an initial inoculum at a 1:1 ratio (**C**) and at a 10:1 ratio (**D**) when ABU 83972 Rif^R^ is released from ELMs. Bars indicate the median. ****P < 0.0001, *t*-test.

ELMs have emerged as a novel approach to modify microbial communities, and we have reported that ABU 83972 within the ELMs multiply and the mechanical forces associated with this proliferation lead to the release of ABU 83972 into the surrounding media (28). Our competition assays, performed with planktonic cells, revealed poor colonization in the murine urinary tract, especially over longer time frames (Fig. 7E and F). Therefore, we tested whether ABU 83972 encapsulated within hydrogels (ELMs) antagonizes UPEC growth in pooled human urine (Fig. 8A). Prior to the competition assay, we assessed the growth of UPEC in pooled human urine with a mock ELM (not encapsulated with ABU Rif^R^). The growth of UPEC was not affected by the presence of mock ELM, indicating that ELM alone does not suppress the growth of UPEC (Fig. 8B). As described in Materials and Methods, ELMs were washed carefully to remove bacteria on the surface of ELMs and to ensure that competition was initiated by ABU 83972 released from the ELM into the pooled human urine. There was a significant increase in ABU 83972 load, which accounted for 75% of the overall population after 24 hours of incubation in pooled human urine, when inoculated in a 1:1 ratio (Fig. 8C; Fig. S8A). When the starting ratio of ABU 83972 to UPEC was increased from 1:1 to 10:1, ELM-released ABU 83972 successfully outcompeted against UPEC and expanded to 82% of the total population by T24 in pooled human urine (Fig. 8D; Fig. S8B). After prolonged incubation, the ABU 83972 population further increased in both conditions, compared to 24 hours, reaching up to 95% of the total population by 72 hours (Fig. 8C and D). Cumulatively, our results reveal that ABU 83972 released from ELMs can successfully outcompete UPEC in pooled human urine.

## DISCUSSION

ABU 83972 is a well-characterized *E. coli* strain that was first isolated from a girl with a history of asymptomatic bacteriuria for at least 3 years (41, 42). ABU *E. coli* strain 83972 confers superior protection against episodes of recurrent UTI in patients (16, 43), representing an alternative approach for preventing UTI. Furthermore, systemic and prolonged antibiotic use is associated with adverse side effects, particularly due to dysbiosis, and an increase in the incidence of infections caused by antibiotic-resistant organisms (44). Developing an antibiotic-independent approach, specifically a human-derived avirulent strain to prevent recurrent UTI, has the potential to minimize the adverse consequences of antibiotic use. Here, we investigated the extent to which ABU 83972 impedes the growth of uropathogens in human urine compared to various laboratory media. Our findings clearly indicate the ability of ABU 83972 to outcompete not only UPEC but also other common uropathogens in pooled human urine.

ABU 83972 is beneficial for UTI prophylaxis due to its avirulent nature and improved adaptation for growth in human urine (42). ABU 83972 lacks the ability to express P and type 1 fimbriae in a functional form (14, 15), decreasing its capacity to adhere to the urothelium, and resists removal of the bacterium by the hydrodynamic forces of urine flow (45–47). However, ABU 83972 persists in the bladder in an adhesin-independent way due to its rapid growth rate in human urine (23). Our findings reveal that ABU 83972 has a generation time of less than 20 minutes in human urine, which enables it to establish in the bladder and overcome population loss that occurs during micturition. ABU 83972 and UPEC CFT073 exhibit comparable growth rates even in diluted pooled human urine and when the strains are diluted 1:1,000 for growth assays. In addition, we found that ABU 83972 and UPEC have a comparable growth rate in pooled human urine even under nutrient starvation, indicating that growth-rate independent mechanisms also allow ABU 83972 to successfully outcompete UPEC.

A previous study has reported that ABU 83972 can outcompete UPEC in pooled human urine (23). Siderophores (48), carbohydrate metabolism (23), and amino acid metabolism (24) are important for the growth of ABU 83972 in human urine. However, the role of these pathways in competition between ABU 83972 and UPEC remains to be evaluated. Comparative genomic analyses have revealed that ABU 83972 and UPEC CFT073 are closely related, with specific mutations in the ABU 83972 genome in the genes encoding adhesins (49). Given these similarities at the genomic level, the ability of ABU 83972 to compete against UPEC remains poorly understood. Our investigation assessed the competitive fitness of ABU 83972 against UPEC as well as other uropathogens and revealed that ABU 83972 successfully competes against a broad range of uropathogens in pooled human urine. A significant finding from our study is that ABU 83972 outcompetes pathogens that are associated with high levels of antimicrobial resistance including *P. aeruginosa*, *A. baumannii,* and *S. aureus*. It is important to note that these organisms are more distantly related to ABU 83972 compared to UPEC and *K. pneumoniae*. Collectively, our findings underscore the importance of determining the mechanistic basis of competition between ABU 83972 and other pathogens as it could unravel novel strategies to inhibit pathogen growth during infection.

Our findings reveal novel features of the nature of competition between ABU 83972 and uropathogens, including outcompeting non-UPEC uropathogens, and are consistent with published reports on ABU 83972 and UPEC competition in human urine (23, 24, 48). Previous studies on the competition between ABU 83972 and UPEC were performed in aerobic conditions. Here, we demonstrate that ABU 83972 effectively outcompetes UPEC regardless of oxygen availability by conducting competition assays under aerobic and anaerobic conditions. To further dissect the role of nutrients in human urine on the competition phenotype, we conducted competition assays in media with varying compositions. We demonstrate that the degree of competition of ABU 83972 against UPEC was higher in pooled human urine than in LB, minimal medium, and artificial human urine. In contrast to human urine, competition between ABU 83972 and UPEC in LB and M9 minimal media results in a reversed pattern of competition where UPEC dominates the population during co-culture with ABU 83972. Furthermore, the competitive fitness of ABU 83972 against UPEC decreases but is not eliminated when urine is diluted.

The competitive advantage of ABU 83972 is retained, and the growth of UPEC is comparable in the spent pooled human urine from ABU 83972, UPEC CFT073, and co-culture of both. This indicates that ABU 83972 is not secreting any metabolites or toxic effectors to inhibit the growth of UPEC. Additionally, our results do not reveal a role for contact dependence in the superior fitness of ABU 83972 relative to UPEC in pooled human urine. Our findings highlight the importance of nutrients found in human urine as key determinants of successful competition between ABU 83972 and UPEC. Another important finding from our study is that the competitive fitness of ABU 83972 is dependent on the cell density. ABU 83972 fails to compete against UPEC when present at a lower density (≤10^3^ CFU/mL) in a 1:1 co-culture in human urine. This observation suggests that the maintenance of a high (at least 10^4^ CFU/mL) and sustained urinary load of ABU 83972 is critical to deterring uropathogen growth in the urinary tract. ELMs, which are essentially functional biofilms embedded with ABU 83972, described in this report could be developed for sustained intravesical delivery of this strain at high levels to deter or combat uropathogens.

A healthy adult human produce ~2 L of urine each day, which reaches the bladder at an average flow rate of 40–80 mL per hour (23). An adult human bladder has a capacity of 300–400 mL, and micturition induces the release of roughly the same volume of urine with none or minimal residual urine (50). We tested whether the constant influx of nutrients and metabolites as fresh urine will impact the competition between ABU 83972 and UPEC. The addition of fresh urine, simulating urinary flow from the kidneys to the bladder, does not impair the ability of ABU 83972 to outcompete UPEC, indicating that the intraspecies competition is not dependent on nutrient limitation in human urine. Therefore, our results implicate that differences in nutrient uptake between ABU 83972 and UPEC might underpin the competitive success of ABU 83972 in urine.

According to a limited number of human trials, inoculation of ABU 83972 is an efficient and safe non-antimicrobial prophylactic against recurrent UTI prevention (19–21, 43, 51). An important observation to emerge from these clinical studies is the high failure rate of colonization, even in patients with incomplete bladder emptying (51). Failure of ABU 83972 colonization and prevention of UPEC and other uropathogen-induced UTI could be attributed to decreased or fluctuations in the urinary load of ABU 83972. Similarly, our *in vivo* study indicated that ABU 83972 showed poor colonization in the murine bladder, resulting in UPEC outcompeting ABU 83972 in adult female CBA/J mice without voiding dysfunction, which is in line with our previous report on ABU 83972 colonization in the murine urinary tract (37). We verified the fitness defect of ABU 83972 in the murine urinary tract using a wild-type and rifampin-resistant isolate. Our findings contrast with that of a previous report where ABU 83972 outcompeted another UPEC strain (NU14) in the bladders of female CBA mice (23). This discrepancy highlights the disconnect between *in vitro* and *in vivo* competition between ABU 83972 and UPEC strains. Additionally, mouse urine is more concentrated than human urine and is not known to support the growth of UPEC *ex vivo* (38). Our study reveals that ABU 83972 has decreased fitness in mouse urine compared to UPEC. Furthermore, ABU 83972 and UPEC face additional bottlenecks in the host due to urodynamic factors and immune effectors shaping the outcome of the competition. Further studies are required to assess if mice are suitable host to investigate interbacterial competition between ABU 83972 and UPEC in the urinary tract.

Poor colonization of ABU 83972 in the urinary tract could be resolved using ELMs. ELMs are hydrogels encapsulating living organisms such as ABU 83972. We have observed that during the proliferation of the embedded cells, the ELMs continuously release those organisms (28). Bladder-resident ELMs might overcome the poor colonization of ABU 83972 due to its sustained release from ELMs to revive the competitive advantage of this strain against uropathogens. In the present study, *in vitro* competition using ELMs yielded promising results, with ABU 83972 released from ELMs outcompeting UPEC in human urine. Our exciting findings represent the first step toward further evaluation of ELMs in the urinary tract for enhanced competition against uropathogens and prevention of UTI.

A limitation of the current investigation is that, although we included multiple uropathogens including two prototypical strains of UPEC (CFT073 and UTI89), ABU 83972 was the sole ABU strain utilized in the competition assays. Even though the model ABU strain *E. coli* 83972 is an effective competitor of uropathogens, additional ABU strains have been identified and characterized (52, 53). Alternative ABU *E. coli* strains such as ABU 61, ABU 106, and ABU 123 have been found to be more competitive than ABU 83972, with low *in vivo* pathogenicity in a murine sepsis model and antibiotic sensitivity (52). We plan to expand our investigations on competitive fitness to other ABU strains, additional UPEC strains, and evaluate the colonization of ABU 83972 in male mice in future studies.

Overall, our study revealed the superior competitive fitness of ABU 83972 against UPEC strains CFT073 and UTI89 and other clinically significant uropathogens in human urine. Future studies are required to determine the mechanisms, including the role of metabolites in human urine and bacterial nutrient uptake systems that enable ABU 83972 to successfully outcompete uropathogens. It is critical to understand the mechanisms involved in the suppression of uropathogens by ABU 83972 to develop novel strategies to combat UTI. Moreover, evaluating ABU 83972-encapsulated ELMs for UTI prophylaxis is an important next step to capitalize on the competitive fitness of this human urinary tract-derived *E. coli* strain with probiotic-like properties.

## Data Availability

All data generated from this study are presented in the main text and supplemental material.

## SUPPLEMENTAL MATERIAL

Supplemental material iai.00173-24-s0001.pdf Figures S1 to S8.

ASM does not own the copyrights to Supplemental Material that may be linked to, or accessed through, an article. The authors have granted ASM a non-exclusive, world-wide license to publish the Supplemental Material files. Please contact the corresponding author directly for reuse.
