# Supplementary material for: Competitive fitness of asymptomatic bacteriuria E. coli strain 83972 against uropathogens in human urine
Source: Infect Immun. 2024 May 23;92(6):e00173-24. doi: 10.1128/iai.00173-24 (PMC11237815; doi:10.1128/iai.00173-24)
Supplement: Supplemental material — Figures S1 to S8. [file iai.00173-24-s0001.pdf]

SUPPLEMENTARY MATERIAL

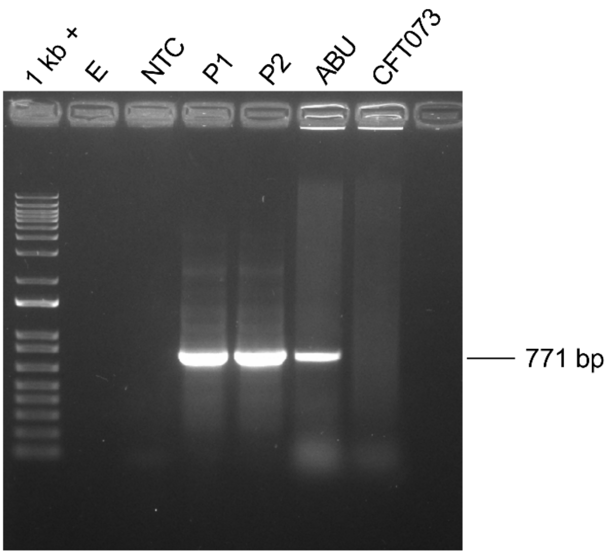

Figure S1: PCR verification of ABU 83972 using pABU primers. 1kb+, DNA ladder; E, empty; NTC, no template control; P1, ABU 83972 plasmid prep biorep 1; P2, ABU 83972 plasmid prep biorep 2; ABU, ABU 83972 Rif^Rovernight culture; CFT073, UPEC CFT073 overnight culture. The expected and observed amplicon size is 771 bp.

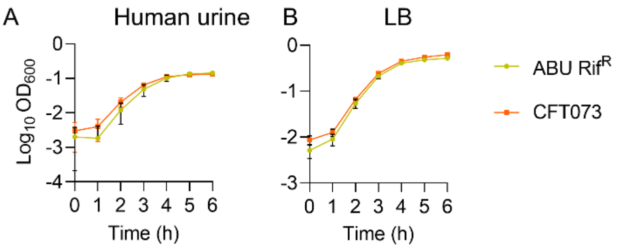

Figure S2: Growth of 1000-fold diluted ABU 83972 and UPEC CFT073 in pooled human urine and LB. Growth pattern of ABU 83972 Rif^R and CFT073 in pooled human urine (A) and LB(B) when diluted 1:1000 at T0. The curves are shown as means of replicates, and error bars indicate standard deviations.

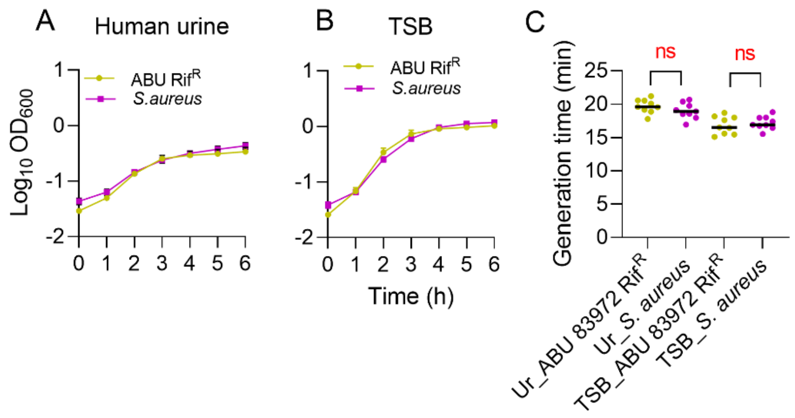

Figure S3. *S. aureus* growth in human urine and TSB. Growth pattern of ABU 83972 Rif^R and *S. aureus* in human urine (A) and in TSB medium (B). The curves are shown as means of replicates, and error bars indicate standard deviations. R studio was used to determine the doubling time of ABU 83972 Rif^R and *S. aureus* in human urine and TSB (C). Bars indicate the median.

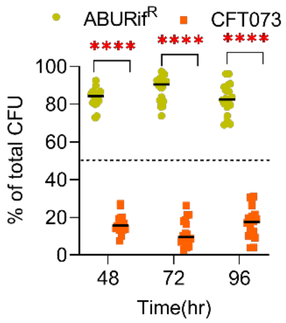

Figure S4. Temporal effects of competition between ABU 83972 and UPEC CFT073. Competition experiment between ABU 83972 Rif^R and CFT073 was continued for longer time frames and CFU was enumerated at 24-hour intervals and the relative abundance was determined. Bars indicate the median. **** $P < 0.0001$, t-test.

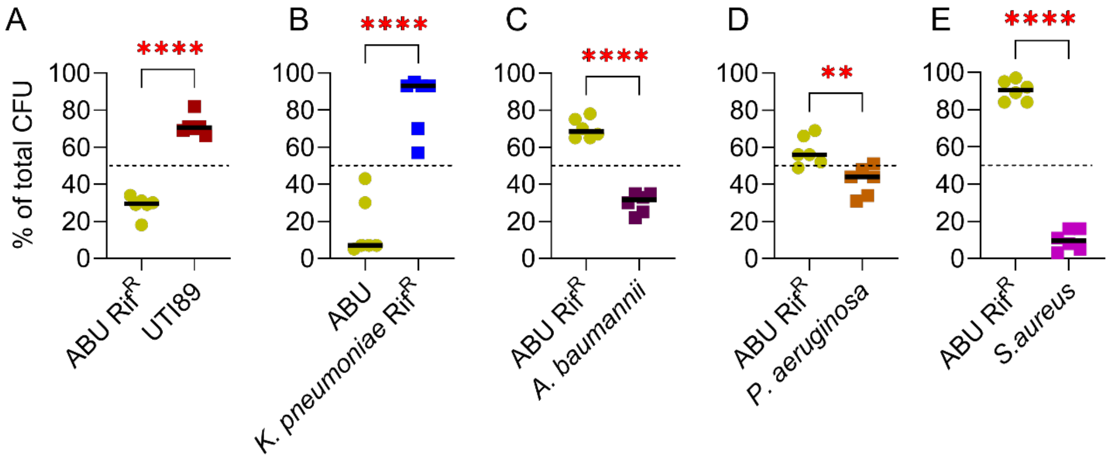

Figure S5. Competition between ABU83972 and uropathogens in rich media. ABU 83972/ ABU 83972 Rif^R was mixed in equal ratio with either UPEC UTI89 (A), *K. pneumoniae* Rif^R (B), *A. baumannii* (C) or *P. aeruginosa* (D) in LB and bacterial load was calculated at T24. Competition experiment between ABU 83972 Rif^R and *S. aureus* was performed in TSB in 1:1 ratio and relative abundance was determined at T24 (E). Bars indicate the median. ** $P < 0.01$, **** $P < 0.0001$, *t*-test.

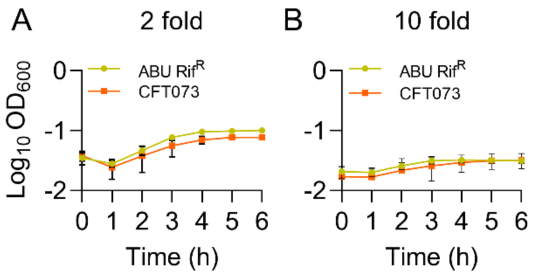

Figure S6: Growth of ABU 83972 and UPEC CFT073 in diluted human urine. The growth pattern of ABU 83972 Rif^{R} and CFT073 in 2-fold (A) and 10-fold diluted human urine (B). The curves are shown as means of replicates, and error bars indicate standard deviations.

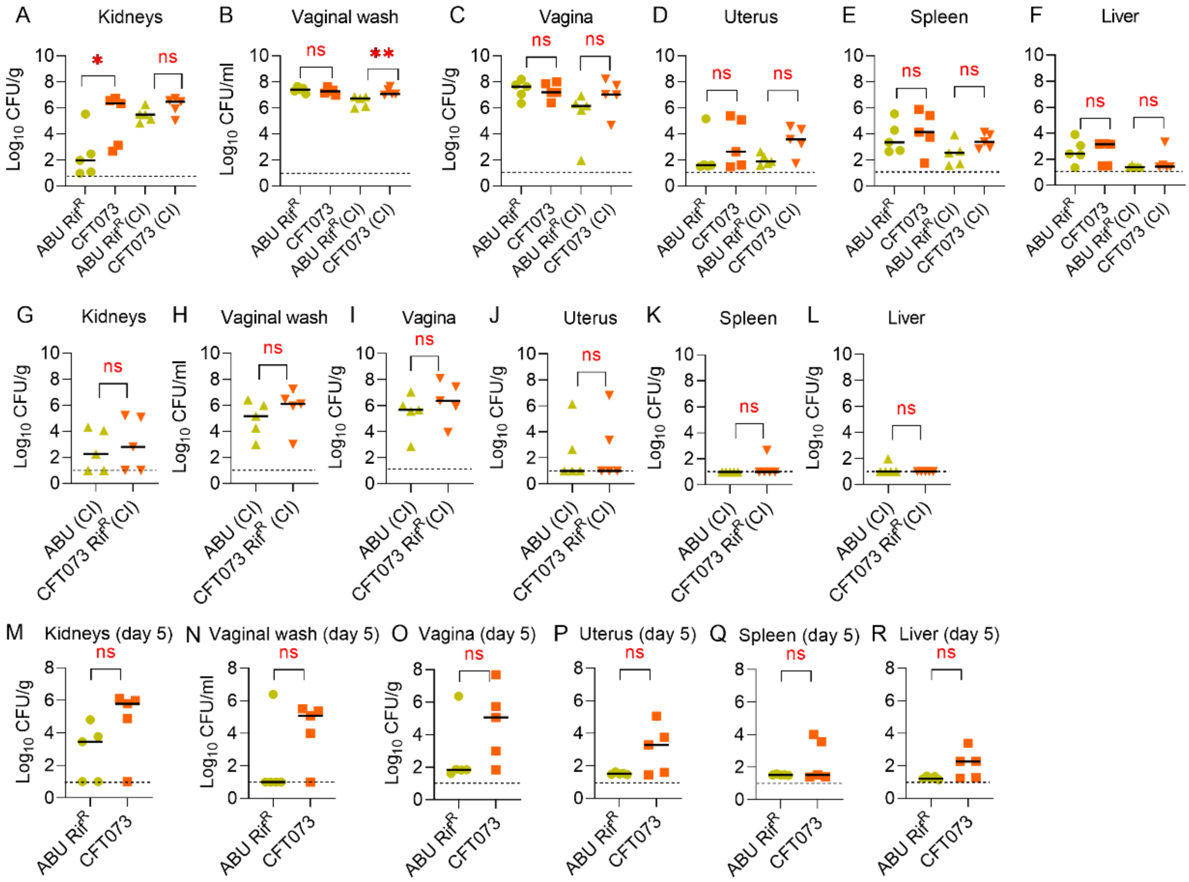

Figure S7. Colonization of ABU 83972 and UPEC in the murine urinary tract. Mixture of ABU 83972 Rif^R and CFT073 (co-inoculation, 1:1 ratio), or ABU 83972 Rif^R alone, or CFT073 alone was inoculated into mice transurethrally. After 24 h, the bacterial load in kidneys (A), vaginal wash (B), vagina (C), uterus (D), spleen (E) and liver (F) was enumerated. G-L: Co-culture of ABU 83972 and CFT Rif^R (1:1 ratio) was inoculated transurethrally and bacterial load in kidneys (G), vaginal wash (H), vagina (I), uterus (J), spleen (K) and liver (L) was determined after 24 hours of infection. M-R: Mice were infected with either ABU 83972 Rif^R or CFT073 and bacterial load in kidneys (M), vaginal wash (N), vagina (O), uterus (P), spleen (Q) and liver (R) was determined at 5 days post-inoculation. Each symbol represents a mouse. Bars indicate the median. Limit of detection was 10 CFU/ml or g. ** $P < 0.01$, *t*-test.

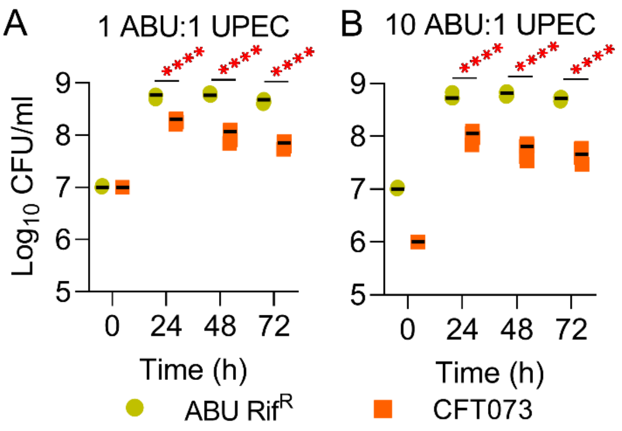

Figure S8. Competition between ELM encapsulated with ABU 83972 *vs* UPEC CFT073. Load of ABU 83972 Rif^R and CFT073 in human urine with an initial inocula at a 1:1 ratio (A) and at a 10:1 ratio (B) when ABU 83972 Rif^R is released from ELMs. Bars indicate the median. **** $P < 0.0001$, *t*-test
